# Evaluating Time Influence over Performance of Machine-Learning-Based Diagnosis: A Case Study of COVID-19 Pandemic in Brazil

**DOI:** 10.3390/ijerph20010136

**Published:** 2022-12-22

**Authors:** Julliana Gonçalves Marques, Luiz Affonso Guedes, Márjory Cristiany da Costa Abreu

**Affiliations:** 1 Department of Informatics and Applied Mathematics, Federal University of Rio Grande do Norte, Natal 59078-970, Brazil; 2 Department of Computer Engineering and Automation, Federal University of Rio Grande do Norte, Natal 59078-970, Brazil; 3 Department of Computing, Sheffield Hallam University, Sheffield S9 3TY, UK

**Keywords:** COVID-19, diagnosis, machine learning, feature importance, eXplainable AI

## Abstract

Efficiently recognising severe acute respiratory syndrome coronavirus 2 (SARS-CoV-2) symptoms enables a quick and accurate diagnosis to be made, and helps in mitigating the spread of the coronavirus disease 2019. However, the emergence of new variants has caused constant changes in the symptoms associate with COVID-19. These constant changes directly impact the performance of machine-learning-based diagnose. In this context, considering the impact of these changes in symptoms over time is necessary for accurate diagnoses. Thus, in this study, we propose a machine-learning-based approach for diagnosing COVID-19 that considers the importance of time in model predictions. Our approach analyses the performance of XGBoost using two different time-based strategies for model training: month-to-month and accumulated strategies. The model was evaluated using known metrics: accuracy, precision, and recall. Furthermore, to explain the impact of feature changes on model prediction, feature importance was measured using the SHAP technique, an XAI technique. We obtained very interesting results: considering time when creating a COVID-19 diagnostic prediction model is advantageous.

## 1. Introduction

The COVID-19 pandemic started in December 2019 and has since continued to spread worldwide without any clear signs of eradication. Many global health systems collapsed at that time due to the large number of people who became infected. The polymerase chain reaction (PCR) test, which detects genetic material from a pathogen or abnormal cell sample, is considered to be the gold standard for diagnosing COVID-19; however, due to the high demand for examinations within short periods of time, PCR tests often end up being delayed and the results are not provided in a timely manner. Although lateral-flow rapid tests provide results more quickly, their availability is often low due to high demand. Thus, using symptoms as a diagnostic tool has a significant advantage: diagnoses can be made quickly, which solves the crucial problem of making timely COVID-19 diagnoses in the context of a pandemic [1,2].

Data classification approaches based on machine-learning (ML) techniques have played an important role [3]. More specifically, on the basis of previous diagnoses of patients who tested positive or negative for COVID-19, supervised learning algorithms built a model that represents the relationship between symptoms (features) and diagnosis through patterns relevant to COVID-19, increasing the probability of COVID-19 identification among other syndromes and illnesses.

However, the emergence of new variants has caused constant changes in the symptoms associate with COVID-19. Because each COVID-19 strain is different, its symptoms can change, are not necessarily the same for all variants, and may not have the same importance in characterising each variant [4,5,6]. Thus, as COVID-19 symptoms vary over time, the features used by machine-learning models to predict the disease also change, directly affecting the performance of machine-learning-based diagnostics. Therefore, it is necessary to observe the impact of these feature changes on classification performance and to recognise them. In addition, since COVID-19 is a respiratory syndrome, its symptoms are very similar to those of other respiratory syndromes, and COVID-19 is often confused with these other diseases [7].

Thus, obtaining an accurate symptom-based model for COVID-19 diagnosis is still a challenge. Here, we propose a machine-learning-based methodology for COVID-19 diagnosis that considers the importance of changes in symptoms over time and, thus, considers the importance of time for model performance. The approach analyses two different time-based strategies for model training.

- In the month-to-month (mm) strategy, the model is trained using the i-th monthly data and validated using the data from the next i + 1-th month.
- The second approach, the accumulated strategy, consists of using data from the months ranging from i = 1 to i + 1 for training, and using the data from month i + 1 + 1 (the month following the period of data accumulation) to validate the model.

In addition, the SHAP approach [8], which is an eXplainable artificial intelligence (XAI) technique, was used to analyse the influence of features on the performance of machine learning models. To validate the proposed method, we used the Xtreme Gradient Boosting (XGBoost) classifier, a decision tree technique based on a gradient-boosting framework. To evaluate the experiments, three classification performance metrics were chosen: accuracy, precision, and recall. COVID-19 cases from Severe Acute Respiratory Syndrome Brazilian datasets were selected as the data.

The remainder of this paper is organised as follows. In Section 2, the literature on symptom-based diagnostics is presented. Section 3 explains the two concepts of XAI and SHAP. Section 4 describes the proposed approach. The experimental setup is described in Section 5. Section 6 presents the results and analysis of the experiments, and Section 7 discusses the findings and future proposals.

## 2. Related Works

Different machine-learning approaches to COVID-19 diagnosis have been reported in the literature, such as those proposed by Khasawneh et al. [9], and Fraiwan et al. [10], who applied convolutional networks to recognise pulmonary diseases related to COVID-19. The first study used a dataset of chest X-ray images from 368 patients with confirmed diagnoses of COVID-19 collected locally and data from three publicly available datasets. Model performance was evaluated four different ways. The results showed high COVID-19 detection accuracy: 98.7%. The second study explored the ability of the model to recognise pulmonary diseases using electronically recorded lung sounds. A dataset of lung sounds collected from 103 patients using a stethoscope, and data from 110 patients from a publicly available challenge database were used. Metrics such as accuracy and precision were used to evaluate the model performance. The results showed that the developed algorithm achieved the highest average accuracy, 99.62%, and precision of 98.85% when classifying patients with pulmonary disease.

Koushik et al. [11] and Devi et al. [12], proposed a methodology to distinguish between positive and negative cases on the basis of symptoms using a public dataset provided by the Israeli Ministry of Health and a variety of machine-learning models with the best accuracy, precision, and recall, over 86%. In the second model, similar experiments were performed using the best model, obtaining 94% accuracy.

Miranda et al. [13] used public data from three states in Brazil (Alagoas, Espírito Santo, and Santa Catarina) and classical machine-learning models and achieved the best accuracy, sensitivity, and specificity, over 85%.

Ahamad et al. [14] and Syed and Khan [15] proposed a methodology to quickly identify COVID-19 using symptoms and a variation of ensembles such as XGBoost. On the other hand, Qomariyah et al. [16] and Gorji et al. [17] explored the symptoms effective for COVID-19 diagnosis, obtaining accuracies of over 75%.

Babu et al. [18] proposed an approach using an ARM-based APRIORI algorithm. A dataset containing 303 cases from the World Health Organisation (WHO) was used. Seven features remained after being discarded by association rules. A support vector machine (SVM), artificial neural networks (ANNs), and random forests (RFs) were used to determine the prediction, accuracy, f1-score, and recall evaluation. The results showed that the APRIORI algorithm achieved the best results, over 97% for both metrics, whereas SVM achieved similar values, over 96% for both.

Zoabi et al. [19], and Arshed et al. [20] both used the SHAP technique and determined that a cough, fever, contact with confirmed cases, the male sex, and age were the most important factors in predicting COVID-19 diagnoses. For classification, similar classical machine-learning models were used, with a prediction performance of 90% for the former and 87% for the latter.

Different from existing work, our work focuses on supervised machine-learning applications for COVID-19 symptom-based diagnosis. Additionally, in most previous work on different machine-learning approaches to COVID-19 symptom-based diagnosis, the features used by the algorithms for prediction are not discussed, and which features are considered to be the most important for building a prediction model of the disease remain to be determined. In the next section, we introduce a feature-importance-based technique that aims to explain the impact of features on model prediction.

## 3. Explainable Artificial Intelligence and SHAP

Increasingly complex problems following the surge in the use of AI to solve real-life problems has resulted in black-box systems being used extensively, particularly in various fields and for different purposes in which the probability of an answer, the reason for the obtained result, and the factors that affect the prediction became increasingly important.

To provide an understandable model of internal operation and prediction, a suite of ML techniques called eXplainable AI (XAI) have been used. To satisfy the psychology of explanation, the goal of these techniques is to maintain a high prediction performance while producing more explainable models, because black-box models often trade explainability for high performance.

Among machine-learning models, models can be categorised as interpretable by design (transparent models), i.e., models that can be explained in human terms and those that can be explained by external techniques (post hoc explainability).

In contrast to black-box models, transparent models, even if the name suggests full transparency, are classified as such only if they can be understood by themselves because transparency is a property. Most models have some degree of interpretability in the domain in which they are interpretable, such as algorithmic transparency, decomposability, and simulatability. Known models, such as k-nearest neighbours (kNN), decision trees, rule-based learning, and Bayesian networks, are considered to be transparent to some degree.

Post hoc explainability is used in models that are not easily interpreted by design. It consists of methods that enhance the interpretability. These techniques can be categorised according to internal model processing: post hoc to shallow models; deep-learning models and convolutional networks; hybrid models that include neural networks and transparent models; and model-agnostic techniques that can be applied to any ML model without considering inner processes. Different post hoc means of explanation, such as text, visualisation, localisation, examples, simplifications, and feature relevance, can be used to improve interpretability.

Feature relevance explanation is one of the most commonly used means of explanation in many machine-learning problems because a very common goal is selecting the most important features of a problem, i.e., the best ones to describe the problem. Many different approaches that assign importance to features to explain the contribution of each feature to the problem description have been proposed. Among them, a technique that is commonly used to explain the prediction of models using the relationship between the output and the features used to produce it was chosen: the Shapley additive explanation (SHAP) is a technique derived from the cooperative game theory Shapley values dating back to the 1950s. The SHAP technique is model-agnostic because it is necessary to compute the values to know only its inputs and outputs without previous knowledge about the model’s internal operation [21]. This characteristic is a defining feature of the concept as it allows for a comparison of input feature values across different model types.

Intuitively, the computation of Shapley values can be explained using the example of a cooperative game. Suppose that there is a set of players (features) with each player contributing to the result of the game (model). If it is possible to determine the total payoff, the average marginal contribution of each player to the result (output) of the game is determined using the Shapley values. Therefore, the Shapley value of a feature can be considered its contribution to the model score [22].

Continuing to use this game as an example, suppose that we have *n* players and val is a function that returns the payoff of the game from a subset of players if only those *n* individuals play. Contribution Φ can then be measured as follows [23]:

(1) $$\Phi_{j}\left(val\right)=\sum_{S\subseteq \{1,\cdots ,p\}\backslash \{j\}}\frac{|S|!(p-|S|-1)!}{p!}\left(val\left(S\cup \left\{j\right\}\right)-val\left(S\right)\right)$$

where *S* is the subset of features used, *p* is the number of features, and $val_{x}\left(S\right)$ is a prediction using the features in *S*.

In summary, the Shapley value is the incremental contribution of each feature to the prediction; it is the weighted average gain that player *j* adds when included in all subsets that exclude *j*.

## 4. Proposed Approach

Considering that the symptoms caused by COVID-19 variants have been changing over time, to maintain the performance of machine-learning-based models for COVID-19 diagnosis, it is necessary to consider this dynamic behaviour in the training strategy of these models. With this in mind, here, we evaluate the performance of two time-based strategies for training machine-learning models for COVID-19 diagnosis: the month-to-month and accumulated time-based strategies.

**Figure 1:**
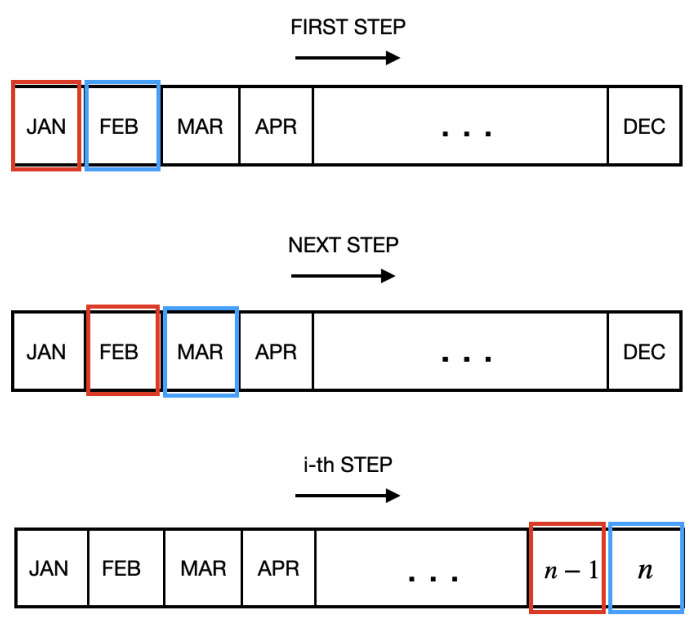
Month-to-month time-based strategy: months highlighted in red indicate training data, and those in blue indicate test data.

In the first training strategy, the month-to-month strategy, the classification model was trained every month using only data from the previous month and tested using data from the current month. Thus, for the i-th month, only data from the i − 1-th month was used to train the machine-learning model, as indicated in Figure 1.

**Figure 2:**
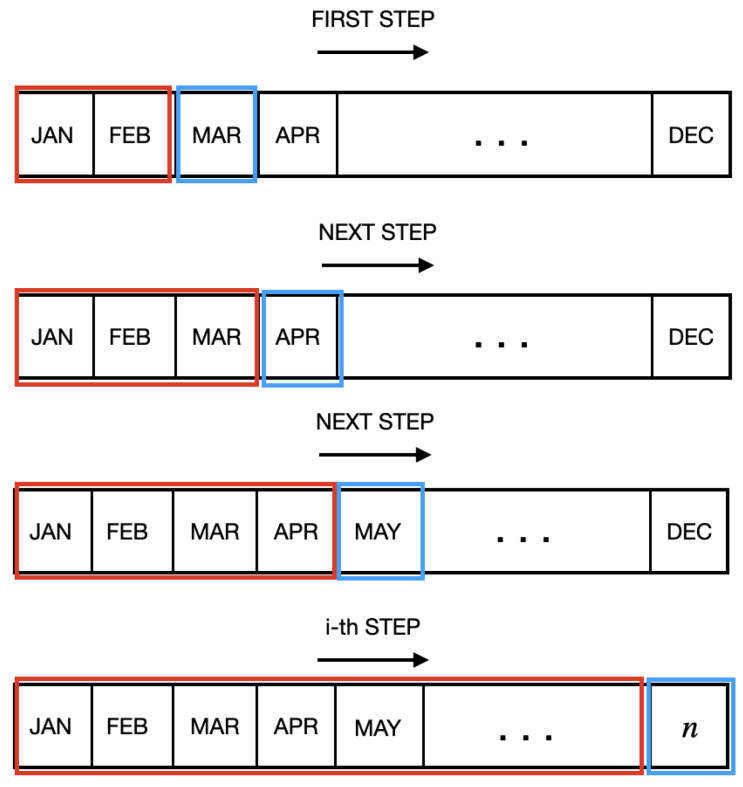
Accumulated time-based strategy: months highlighted in red indicate training data, and those in blue indicate test data.

The second training strategy, the accumulated strategy, trained the model every month using the data from the first month to the previous month in the database. Thus, for the i-th month, the training dataset consisted of data from the months ranging from i = 1 to i − 1 and data from the i-th month was used for test data, as shown in Figure 2.

Other contributions of this work include an analysis of how the symptoms change over time and how to better evaluate the model performance. In this way, the SHAP XAI-based technique was used to explain how each feature (symptom) influences the machine-learning model performance over time, and the accuracy, precision, and recall performance metrics were considered to evaluate the performance of the machine-learning models.

## 5. Materials and Methods

**Figure 3:**
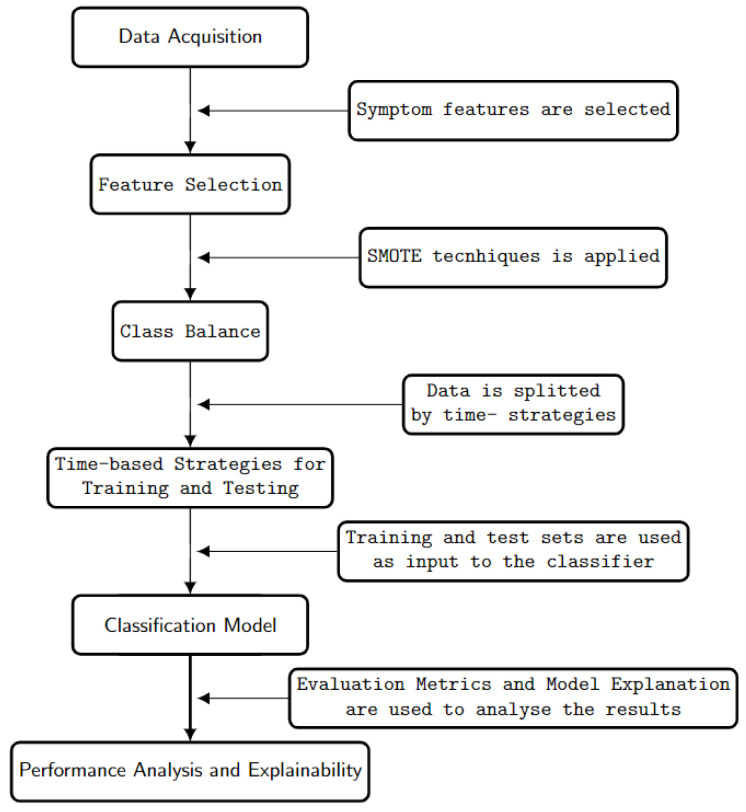
Proposed approach.

This section presents the process followed when performing the experiments, also shown in Figure 3, which consists of six steps: data acquisition, in which the data were chosen; feature selection, in which the features suitable for the problem were selected; class balance, which was performed on the samples to prepare them for classification; time-based training–test process, in which the data were split according to time-based strategies, and the training and test sets were selected; classification, in which the chosen classifier was used for prediction; lastly, results and analysis, in which evaluation metrics were used to measure the performance of the model prediction, and a feature importance technique was used to explain the model output.

### 5.1. Data Acquisition

As data for the experiments, the 2020 and 2021 datasets for severe acute respiratory syndrome syndrome (SARS) obtained from openDataSUS [24], a publicly accessible database regarding the Brazilian health situation, were used. These datasets were chosen because they had the highest number of infections and deaths across the country, rendering the data a significant source of information for testing our hypotheses.

The datasets contain social demographics, symptoms, risk factors, comorbidities, and laboratory findings for all states.

### 5.2. Feature Selection

Because the disease impacted distinct regions of the country differently during the same time period, São Paulo (SP) state data were chosen as the most representative COVID-19 dynamics in the country. The dataset contains information on 652,498 individuals. Next, we describe the 12 features used in the experiments.

1. DESC_RESP (RESP_DIS) indicates whether the patient presented with respiratory distress.
2. DIARREIA (DIARRHOEA) indicates whether the patient presented with diarrhoea.
3. DISPNEIA (DYSPNEA) indicates whether the patient presented with dyspnoea.
4. DOR_ABD (ABD_PAIN) indicates whether the patient presented with abdominal pain.
5. FADIGA (FATIGUE) indicates whether the patient presented with fatigue.
6. FEBRE (FEVER) indicates whether the patient presented with fever.
7. GARGANTA (THROAT) indicates whether the patient presented with a sore throat.
8. PERD_OLFT (LOSS OF SMELL) indicates whether the patient presented with a loss of smell.
9. PERD_PALA (LOSS OF TASTE) indicates whether the patient presented with a loss of taste.
10. SATURACAO (SATURATION) indicates whether the patient presented with low oxygen saturation (O${}_{2}<$ 95%).
11. TOSSE (COUGH) indicates whether the patient presented with a cough.
12. VOMITO (VOMIT) indicates whether the patient presented with vomiting.

### 5.3. Class Balance

Class imbalance is a significant issue in the datasets used. Commonly, classes of problems have fewer examples than other classes do. However, most classifiers tend to assume that the classes in a dataset are balanced and biased toward the class, with more examples of the majority class having partial performance in the class with fewer examples (minority class), which is usually the most significant [25].

In this problem, the minority class (CLASSI_FIN = 1) indicates a positive COVID-19 diagnosis, whereas the majority class (CLASSI_FIN = 0) indicates a negative diagnosis. To mitigate this issue, the synthetic minority oversampling technique (SMOTE) was used to oversample examples from the minority class. For the experiments, we use *k* = 4 and *sampling_strategy* = 0.7, which is the sampling used to resample the minority class.

### 5.4. Time-Based Strategies for Training and Testing

This step consists of applying two time-based strategies for training and testing, as mentioned before.

### 5.5. Classification Model

To classify the data, a machine-learning technique frequently applied to different COVID-19 diagnostic approaches was used [16,26]. The Xtreme Gradient Boost (XGBoost) is an algorithm based on the gradient-boosting framework. It is an optimised library designed to solve different ML problems and is considered fast, accurate, and highly efficient. The algorithm was used as a classifier (XGBClassifier) and was found in Python’s scikit-learning library [27]. The algorithm was trained using default hyperparameters.

### 5.6. Performance Analysis and Explainability

The accuracy, precision, and recall classification metrics [28] were used to evaluate the models and are described here:

- Accuracy is the percentage of samples correctly predicted by the classifier in terms of the total number of predictions.
  (2) $$Accuracy=\frac{TP+TN}{TP+TN+FP+FN}$$
- Precision refers to the percentage of true positive samples in relation to the total number of samples classified as positive.
  (3) $$Precision=\frac{TP}{TP+FP}$$
- Recall is the percentage of true positive cases among those expected to be true, i.e., *TP* and *FN*.
  (4) $$Recall=\frac{TP}{TP+FN}$$

*TP* represents the true positive cases, *FP* represents the number of false positive cases, *TN* represents the true negative cases, and *FN* represents the number of false negative cases.

In addition, to perform model explainability, the feature importance technique SHAP was used, which is found in the SHAP library [29] for the Python language.

## 6. Results and Analysis

In this section, we present the results obtained using the two time-based strategies proposed to train the machine-learning models. In addition, the obtained results were analysed using graphics from SHAP, an XAI-based Python package. In the experiments, we used data collected from January 2020 to November 2021.

### 6.1. Results

**Figure 4:**
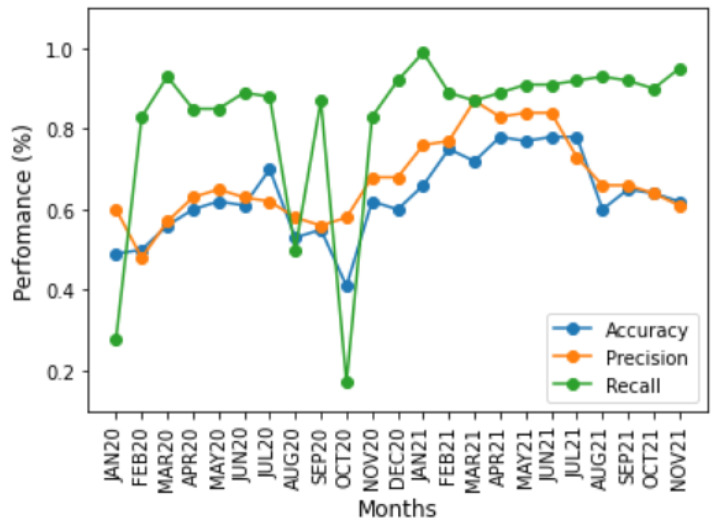
Performance of classification metrics vs. month-to-month strategy.

**Figure 5:**
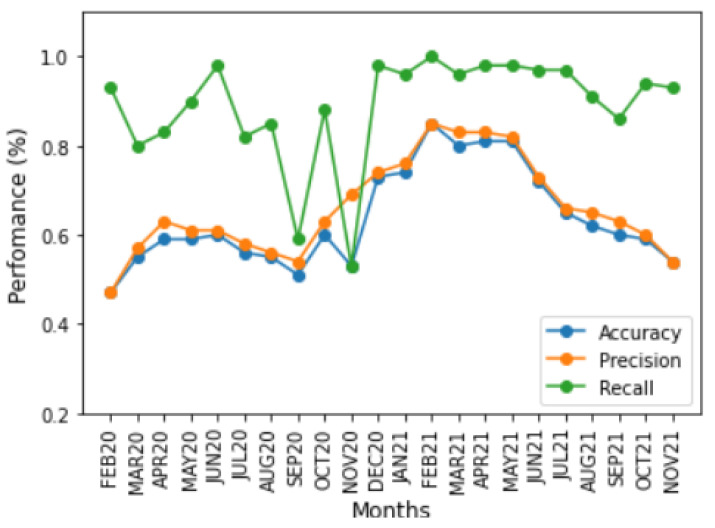
Performance of classification metrics vs. accumulated strategy.

Accuracy, precision, and recall were used as performance metrics [28] to evaluate the performance of the two time-based training strategies, the month-to-month and accumulated strategies. Figure 4 and Figure 5 show the impact of changes in the symptoms on model performance over time using these three performance metrics for the two time-based training strategies. Although the model’s performance was low for most time periods, there were periods of high performance, especially from January to June, in both years. In addition, the performance of the metrics between time periods showed the impact of sudden changes in COVID-19 symptoms. For all graphics, the *x* axis corresponds to the period of time in months for each approach, and the *y* axis corresponds to the performance of each metric in percentage (%).

The classification used by the Brazilian public health system to label SARS cases classifies COVID-19 cases as positive or unspecified SARS (cases in which no other etiological agent was identified; it was not possible to collect/process clinical samples for laboratory diagnosis, or to confirm by clinical–epidemiological criteria, clinical imaging, or clinical diagnosis). However, it is not possible to separate samples that are negative for COVID-19 from other sample types, which hinders distinguishing each type of case using this classification.

The accuracy and precision metrics had similar results, tending towards 50%, because the base was balanced. However, as a consequence of database labelling, the few *TN* cases and nonexistent *FP* cases decreased the accuracy, whereas precision was only affected by *FP* cases; the metric only considered *TP* cases. This is evident from the results shown in Figure 4 because the training and test sets are different. However, for the results shown in Figure 5, *TN* and *FN* accumulated cases increased the accuracy, becoming more similar to precision.

On the other hand, the recall metric presented different symptom dynamics because this performance metric only measured *TP* and *FN* cases when symptoms did not change. Thus, the classifier tended not to identify *FN* cases because of mislabelling, resulting in high performance. However, when symptoms change from one period to another, there may be an increase in the *FN* evaluation. *FN* cases affected performance according to the significance of the symptom change. In the month-to-month strategy for the OCT20 period, where the symptoms substantially changed related to SEPT20, decreasing the recall, the same occurred with NOV20, where the symptoms were more important in identifying a case related to OCT20, increasing the performance. In the month-to-month approach. These dynamics are apparent because the training and test datasets are different, making it possible to detect changes in symptoms of the COVID-19. In the accumulated approach, the accumulation of information dampened sudden changes in performance because the same symptoms appeared in all the training sets.

### 6.2. Model Explanation

Waterfall plot SHAP graphics were chosen for the model explanation. These figures show the contribution of each feature to the final prediction. On the *y axis*, the features used by the model are indicated. At the bottom, the *x axis* is the base value (E[f(x)]), and the average number of predicted cases for these samples and the top x axis are the ending values f(x), which is the predicted number of cases for the samples used. The bars in the left direction indicate a negative impact (−) on the prediction, whereas the bars in the right direction indicate a positive impact (+). In addition, blue indicates low impact, and pink indicates high impact. Each graph was plotted using 1000 samples.

To understand the impact of symptom changes on COVID-19 prediction, SHAP graphs were plotted for some of the periods when the performance changed significantly, specifically, a shift in the peaks, because in these periods, the changes in symptom importance were more evident.

**Figure 6:**
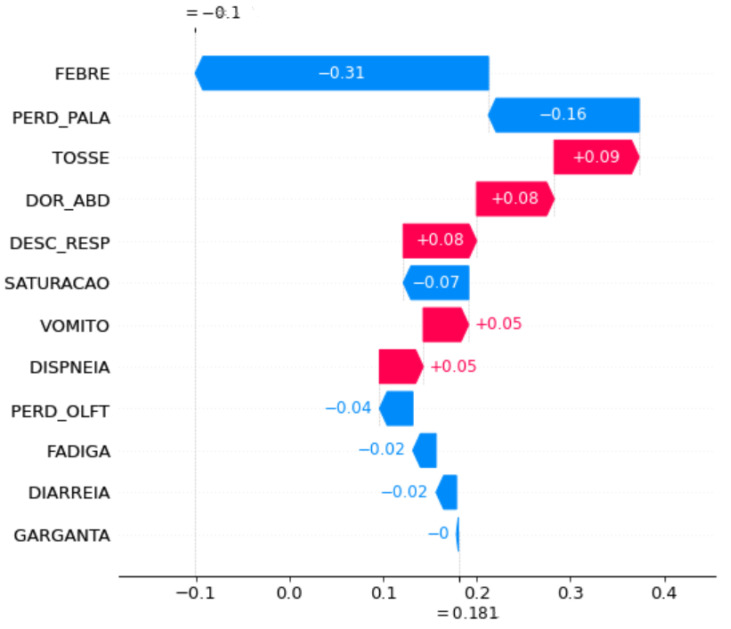
Feature importance for the period of OCT20 using the month-to-month strategy.

**Figure 7:**
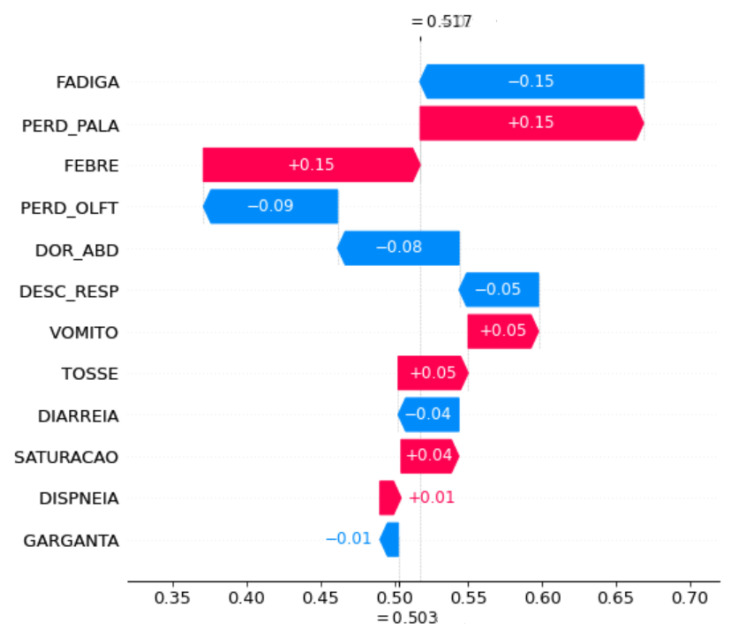
Feature importance for the period of NOV20 using the month-to-month strategy.

Figure 6 and Figure 7 show the feature importance of the periods OCT20 and NOV20 in the month-to-month strategy. Initially, symptoms that had occurred in OCT20 contributed to a decrease in prediction accuracy, whereas the features of NOV20 increased the prediction accuracy, which was clear from the initial and final prediction values. For OCT20, the prediction started 0.18% after the contribution of the features. Subsequently, it decreased to 0.10. For NOV20, the prediction started at 0.50%, and after adding features, it increased to 0.51%.

These features also did not have the same importance. For example, on OCT20, FEBRE contributed the most to the identification of a case, while on NOV20, FADIGA contributed the most. Both decreased performance. The PERD_PALA feature appeared to be the second most important feature that affects prediction for both months. For OCT20, it decreased the prediction accuracy, whereas for NOV20, it increased prediction accuracy. VOMIT was the only feature that contributed equally to prediction for both months.

**Figure 8:**
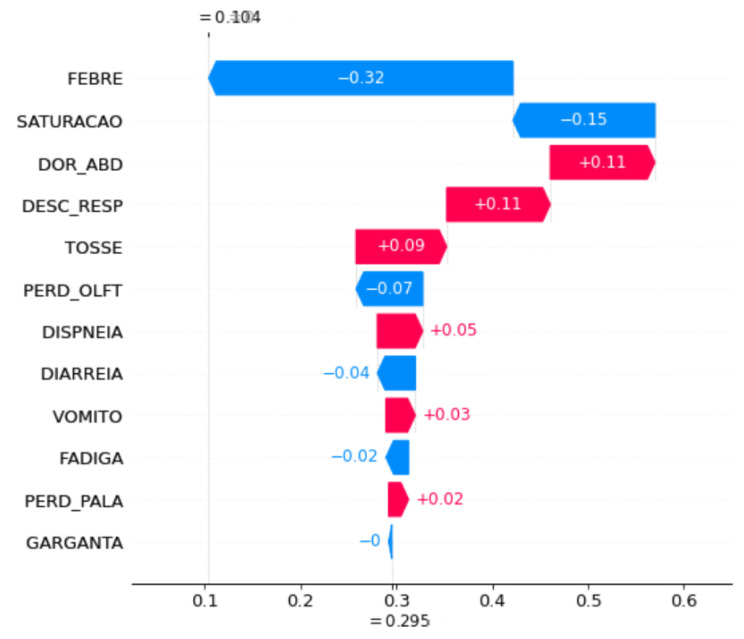
Feature importance for the OCT20 period using the accumulated strategy.

**Figure 9:**
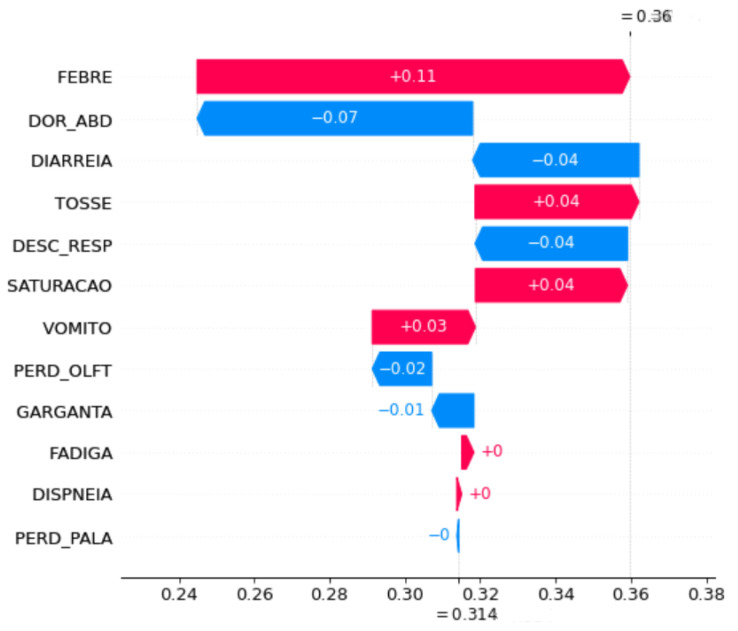
Feature importance for the NOV20 period using the accumulated strategy.

Figure 8 and Figure 9 show the feature importance of the OCT20 and NOV20 periods in the accumulated strategy. The most important feature for OCT20 (FEBRE) using the accumulated strategy decreased the prediction accuracy. It started at 0.29%, and after adding features, it decreased to 0.10%, whereas for NOV20, the features increased the prediction accuracy, increasing the prediction rate from 0.31% to 0.36%. In addition, for OCT20, this feature contributed negatively; however, for NOV20, it contributed positively. Another important aspect to be emphasised is that the second most important feature for OCT20 is SATURACAO, whereas that for NOV20 is DOR_ABD, but both have a negative impact on model prediction. Additionally, unlike for the previous strategy, GARGANTA has an unimpressive contribution.

Lastly, except the FEBRE and FADIGA features for the accumulated strategy and the VOMITO feature in the month-to-month strategy, the features from one period to another did not have the same order of importance and often contributed to prediction accuracy differently, increasing or decreasing the accuracy of prediction by different percentages. This significant difference between the symptoms that characterise the cases results in errors in prediction, since a case is identified as *TP* in a given period and in the subsequent period, the features are not the same, labelling the case as *FN*.

## 7. Conclusions

In this study, two time-based strategies were proposed and analysed to obtain accurate machine-learning-based models for COVID-19 diagnosis considering changes in symptoms over time. These two time-based strategies were the month-to-month and accumulated strategies. Both used different training and test datasets to obtain COVID-19 diagnostic models. This approach proved to be more realistic and allowed for the importance of changes in COVID-19 symptoms over time to be determined. More specifically, the results show that the recall performance metric was more efficient than the accuracy and precision performance metrics in detecting changes in symptoms. Thus, the recall metric was able to identify variations in symptoms that had been classified as *FN*, indicating that it is better suited for data analysis of the changes over time. However, mislabelling affects the accuracy and precision metrics. Because there are few to nonexistent *TN* and *FP* cases, accuracy is most affected by *FN* and *TP* cases, whereas precision is affected only by *TP* cases, which renders the performance metrics similar for both approaches. Moreover, the XAI technique was adequate for showing symptom changes and their impact on the model predictions.

Consequently, to improve the prediction of this type of data, it is important to consider techniques that seek to improve performance while considering the issue of mislabelling. As an improvement to this study, semisupervised machine-learning and incremental-learning techniques such as incremental DBSCAN should be considered.

## Data Availability

The data used in this work are available in OpenDataSUS (https://opendatasus.saude.gov.br/, accessed on 2 May 2022), a database maintained by Brazil Ministry of Health. The code developed to perform the experiments is available at https://github.com/JuhCrln/COVID-19-Supervised- (accessed on 7 December 2022).

## Abbreviations

The following abbreviations are used in this manuscript:

SARS-CoV-2	Severe acute respiratory syndrome coronavirus 2
XGBoost	Xtreme Gradient Boost
SHAP	SHapley Additive explanations
XAI	eXplainable AI
PCR	Polymerase chain reaction
ML	Machine learning
ARM	Association rule mining
WHO	World Health Organisation
SVM	Support vector machine
ANN	Artificial neural network
RF	Random forest
kNN	k-nearest neighbours
SP	São Paulo
DESC_RESP (RESP_DIS)	Desconforto Respiratório (Respiratory Discomfort)
DOR_ABD (ABD_PAIN)	Dor Abdominal (Abdominal Pain)
PERD_OLFT	Perda de Olfato (Loss of Smell)
PERD_PALA	Perda de Paladar (Loss of Taste)
CLASSI_FIN	Classificação Final (Final Classification)
SMOTE	Synthetic minority oversampling technique
XGBClassifier	Xtreme Gradient Boost Classifier
*TP*	True positive
*TN*	True negative
*FP*	False positive
*FN*	False negative
SEPT20	September 2020
OCT20	October 2020
NOV20	November 2020
